# Clinical outcomes of arthroscopic all-inside anterior talofibular ligament suture augmentation repair versus modified suture augmentation repair for chronic ankle instability patients

**DOI:** 10.1186/s12891-023-07085-3

**Published:** 2024-01-10

**Authors:** Dahai Hu, Nan Wang, Huajun Wang, Dongyi Fan, Qiang Teng, Xiaofei Zheng, Huige Hou

**Affiliations:** 1 Department of Bone and Joint Surgery and Sports Medicine Center, The First Affiliated Hospital, Jinan University, Guangzhou, 510630 Guangdong China; 2 International School, Jinan University, Guangzhou, 510632 Guangdong China; 3 Department of Clinical Medicine, School of Medicine, Jinan University, Guangzhou, 510632 China

**Keywords:** Anterior talofibular ligament, Chronic ankle instability, Augmentation repair, Modified suture augmentation repair, Outcomes, Arthroscopy

## Abstract

**Background:**

To compare the clinical efficacies of arthroscopic anterior talofibular ligament suture augmentation repair and modified suture augmentation repair in patients with chronic ankle instability (CAI).

**Methods:**

From October 2019 to August 2020, 100 patients with CAI were enrolled after propensity score matching analysis and observed for two years. Among them, 50 underwent modified suture augmentation repair and the other 50 underwent suture augmentation repair. The clinical efficacies of CAI treatments were evaluated using the American Orthopedic Foot and Ankle Society (AOFAS) clinical rating scale, visual analog scale (VAS), and anterior drawer test scores.

**Results:**

The postoperative AOFAS score of the modified suture augmentation repair group (83.8 ± 11.3) was significantly higher than that of the suture augmentation repair group (76.3 ± 11.3; *P* = 0.001). The VAS (*P* = 0.863) and anterior drawer test (*P* = 0.617) scores were not significantly different between the two treatment groups.

**Conclusion:**

Both the modified suture augmentation repair and suture augmentation repair demonstrated good clinical efficacies. The AOFAS score of the modified suture augmentation repair group was superior to that of the conventional suture augmentation repair group. Thus, modified suture augmentation repair is a feasible and practical surgical technique for CAI treatment.

**Supplementary Information:**

The online version contains supplementary material available at 10.1186/s12891-023-07085-3.

## Introduction

Chronic ankle instability (CAI) is a perception of “giving way,” usually resulting from laxity or injury of ligaments around the ankle joint [1]. Moreover, the damage of anterior talofibular ligament (ATFL), which is characterized by mechanical instability of the ankle joint, is an important cause of CAI that interferes with the activities of daily living [1–4]. Approximately 40% of patients with untreated ankle sprains later develop CAI [5]. Currently, the management of CAI involves both surgical and nonsurgical treatments [6]. Nonsurgical treatments mainly comprise physical therapy and taping [7]. However, surgical treatments have better outcomes than nonsurgical treatments in patients with CAI [6]. Therefore, several modified surgical techniques have been described earlier owing to the challenges faced by orthopedic surgeons in choosing an appropriate surgical technique for the management of patients with CAI [8–11].

In 2018, Vega et al. were the pioneers in using the ATFL suture augmentation repair technique via ankle arthroscopy to treat patients with CAI and achieved good clinical outcomes [12]. However, suture augmentation repair is not applicable in all cases [13]. In clinical practice, we have found that stitching the upper and lower tracts of the ATFL into a single unit allows the forces of traction to be transferred from the ATFL to the calcaneofibular ligament (CFL). This greatly enhances the stability of the repaired ATFL. We refer to this method as the modified suture augmentation repair technique, which has been applied in the treatment of patients with CAI. Compared to modified suture augmentation repair, suture augmentation only repaired the damaged upper or lower tracts of the ATFL. At present, studies on the clinical efficacy of these two surgical schemes are scarce, and an objective evaluation is lacking.

Therefore, in this study we aimed to compare the clinical efficacy of arthroscopic suture augmentation repair and modified suture augmentation repair to provide a guideline for orthopedic surgeons to choose appropriate surgical techniques. Additionally, it has been hypothesized that modified suture augmentation repair may achieve better clinical outcomes than conventional suture augmentation repair.

## Materials and methods

This single-center retrospective study aimed to evaluate the clinical efficacies of suture augmentation and modified suture augmentation repairs.

We included 103 patients out of 196 inpatients with orthopedic disorders who visited our hospital between October 2019 and August 2020. Three patients, including those with arthritis (n = 2) and systemic disease (n = 1), were excluded.

Before performing surgeries, data pertaining to all participants were collected after obtaining written informed consents, according to the principles of the Declaration of Helsinki. Subsequently, propensity score matching (PSM) was applied, and a logistic regression model was used to achieve a balanced group at baseline. Age, sex, body mass index (BMI), preoperative American Orthopedic Foot and Ankle Society (AOFAS) score, and preoperative anterior drawer test grade were the final covariates. The PSM ratio was 1:1 with a caliper width of 0.05. Ultimately, 100 patients with CAI (50 patients who underwent suture augmentation repair and 50 patients who underwent modified suture augmentation repair) were included in this study. The surgical technique was decided by the same senior orthopedic surgeon.

### Inclusion and exclusion criteria

The inclusion criteria were as follows: (1) patients with a history of CAI or ankle sprain in the past 6 months who were unresponsive to conservative treatment; (2) age < 60 years; (3) patients with no previous history of ankle surgery; and (4) stress radiographic evaluation findings showing that the difference in the talar tilt angle was 10° and the absolute talar tilt angle was 15° between the two ankle laxities. The exclusion criteria were as follows: (1) patients with ankle osteoarthritis or anatomical deformities such as sepsis, rheumatoid arthritis, and tuberculosis arthritis; and (2) patients with pre-existing medical conditions such as systemic or neuromuscular diseases or obesity that affected prognosis.

### Operative techniques

#### Modified suture augmentation (MSA) repair

The patient was placed under general anesthesia before the surgery, and the ankle joint was placed in the dorsiflexion and lateral decubitus positions.

The standard anteromedial (at the distal end of the ankle line and close to the lateral side of the third peroneal muscle tendon), anterolateral (at the level of 0.5 cm at the distal end of the ankle line and close to the lateral side of the third peroneal muscle tendon), and accessory anterolateral portals (at the level of 0.5 cm at the distal end of the ankle line, close to the anterior side of the fibula and 1.0 cm away from the fibular tip) were carefully established to prevent damage to the superficial peroneal nerve.

A cannula was inserted through the anterolateral portal, and an arthroscope (Arthrex, 28,731 BWA, 4.0 mm) was used to visualize the structure of the articular cavity. Subsequently, the lateral gutter was exposed, and the lateral articular capsule was opened. A slim guide needle was used first, and a No. 0 non-absorbable suture (Smith & Nephew, Arthrex) was folded in half using a lumbar puncture needle (Zhejiang Runqiang Medical Instrument, 17G). The lumbar puncture needle was then passed through the inferior fascicle of the ATFL from the outside to inside. Next, using an arthroscopic gripper, No. 0 suture was captured through the accessory portal, where the folded suture ended to form a ferrule. A thread grabber (Johnson 214,626) was used to pull both ends of the suture out of the ferrule and then out of the accessory portal. It could be observed that the ligament was tightly grasped by the suture. Subsequently, a knotless anchor (Pushlock 2.9 mm x 15 mm, Arthrex) was introduced through a suture passer, and the ligament was repaired.

**Fig. 1:**
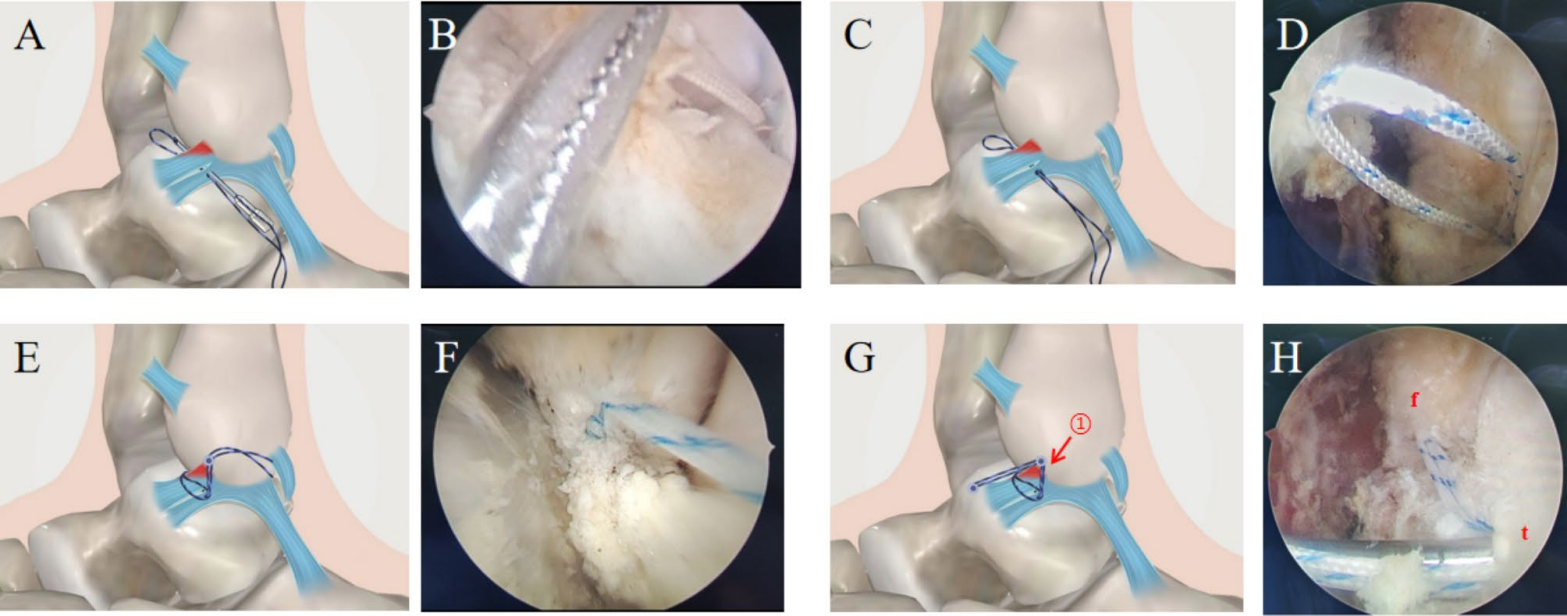
The operation diagram for modified suture augmentation repair. **A**, **B**, **C**, **D**: The No. 0 non-absorbable suture was introduced through the inferior fascicle of ATFL; E, F, G, H: The knotless anchor was introduced, and ATFL was repaired. ATFL: Anterior Talofibular Ligament. f: fibula; t: talus; ①: suturing the upper and lower tracts of the ATFL as a whole

During anchoring, drilling at the center of the ATFL attachment was performed by employing safety insertion angles of 30° from the fibular longitudinal axis. The sutures were threaded through an anchor. After the anchors were implanted, both ends were sutured without cutting. The drill guide was inserted through the anterolateral portal and placed at the center of the talar neck to avoid invasion of the subtalar joint. The hole was drilled, and the bone anchor with the suture was passed through the portal vein and introduced into the cavity by impaction. Finally, the suture ends were cut, and the incision was closed. The operational diagram is shown in Fig. 1.

#### ATFL suture augmentation (SA) repair

**Fig. 2:**
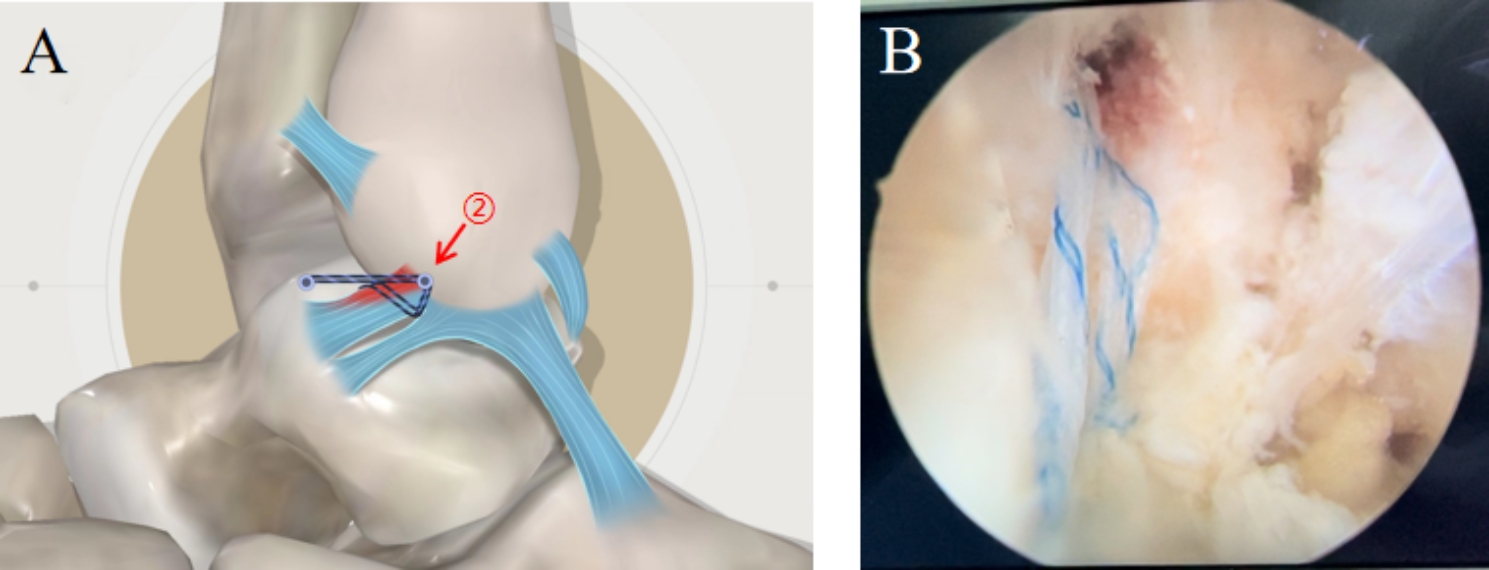
The operation diagram for suture augmentation repair. **A**: The operation diagram. **B**: Arthroscopic operation. ②: only repaired the damaged upper tract of ATFL

Except for the opening of the lateral joint capsule, all surgical procedures were similar to those of modified suture augmentation repair. Therefore, the lumbar puncture needle was passed through the superior fascicle of the ATFL from the outside to inside. The operational diagram is shown in Fig. 2.

### Postoperative rehabilitation

A non–weight-bearing, short-leg cast was applied on the ankle in a neutral position. After two weeks, the cast was replaced with a controlled ankle movement (CAM) boot. A gradual physical therapy program involving low-impact ankle range of motion and strengthening was initiated. Depending on the progress, the supportive boot was removed after 4–6 weeks and the patient was allowed to return to normal activities of daily living.

### Clinical assessment

The patients returned to the hospital for follow-up at 1, 3, 6, 12, and 24 months postoperatively, and the clinical outcomes at the last follow-up were recorded.

#### The American orthopedic foot & ankle society (AOFAS)

The AOFAS is primarily used to evaluate the functional status of the feet and ankles. It has a total score of 100 points and comprises three subscales: pain, function, and alignment [14]. Overall, a score of 90–100 points is considered “excellent,” a score of 80–90 points is considered “good,” a score of 60–80 points is considered “fair,” and a score of < 60 points is considered “bad” [14].

#### Visual analog scale (VAS)

The VAS was used to assess the pain status of the patients. It consists of 0–10 points, 0 points for no pain, and 10 points for severe pain [15, 16].

#### Anterior drawer test

The anterior drawer test is one of the methods used to evaluate ankle instability in patients. While performing the test, the patient is seated with the lower leg hanging over the edge of the examination bed. The doctor stabilizes the patient’s distal tibia with one hand and applies an anterior force to the calcaneus with the other hand [17]. It is mainly divided into four grades: Grade 0 (translation is less than 5 mm compared with the opposite side), Grade 1 (translation 5–10 mm), Grade 2 (translation 10–15 mm), and Grade 3 (translation > 15 mm) [18].

### Statistical analysis

SPSS (IBM, Armonk, NY, USA) and GraphPad Prism (GraphPad Software, San Diego, CA, USA) were used for the data analysis. The t-test was used to compare the clinical outcomes of age, follow-up time, AOFAS scores, and VAS scores. The chi-squared test was used to analyze the anterior drawer test scores. The normality of the distribution was evaluated using the Shapiro–Wilk test. PASS (PASS package, NCSS, USA) was used for the power analysis. The bilateral αvalue was 0.05, sample size was 100 and the test efficacy was 90%. In our study, a *P*-value < 0.05 was considered statistically significant.

## Results

**Fig. 3:**
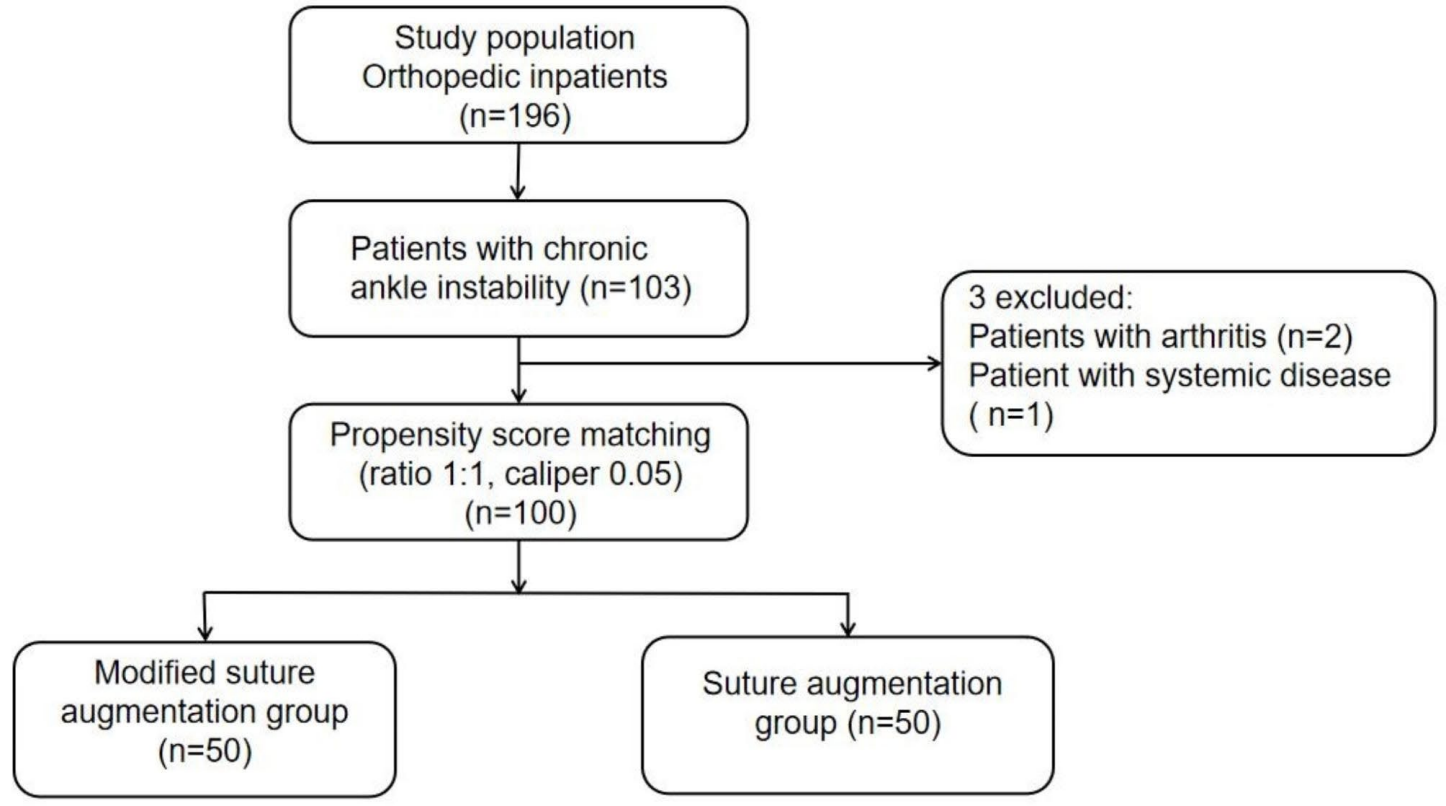
Trial profile

One hundred patients (59 men and 41 women) from October 2019 to August 2020 were included in the study after PSM (Fig. 3). Among them, 50 patients underwent suture augmentation repair (29 men and 21 women, follow-up duration: 24.3 ± 2.0 months), and the other 50 underwent modified suture augmentation repair (30 men and 20 women, follow-up: 24.2 ± 1.9 months). The two groups were comparable after PSM validation. There were no statistically significant differences in age, follow-up time, BMI, preoperative AOFAS score, preoperative VAS score, or preoperative anterior drawer test scores between the two groups (*P* = n.s.). The baseline characteristics of these groups are presented in Table 1.

**Table 1:** Baseline characteristics of patients included

Variable	SA group	MSA group	t/X2 value	P value
Number of patients	50	50		
Age, years	33.1 ± 7.9	32.3 ± 7.6	0.476	0.535
Sex (M/F), n	29/21	30/20		
Follow-up, months	24.3 ± 2.0	24.2 ± 1.9	0.123	0.962
Body Mass Index	23.6 ± 2.7	23.2 ± 2.4	0.233	0.791
AOFAS	65.2 ± 9.8	65.9 ± 11.1	0.344	0.593
VAS	2.5 ± 1.0	2.6 ± 1.0	0.489	0.526
Anterior drawer test, %			0.154	0.917
Grade 0	0(0%)	0(0%)		
Grade 1	5(10%)	3(6%)		
Grade 2	30(60%)	30(60%)		
Grade 3	15(30%)	17(34%)		

Data are number of patients n (%) or mean ± SD

AOFAS: The American Orthopedic Foot & Ankle Society; VAS: Visual analog scale; SA: Suture Augmentation Repair; MSA: Modified Suture Augmentation Repair

**Fig. 4:**
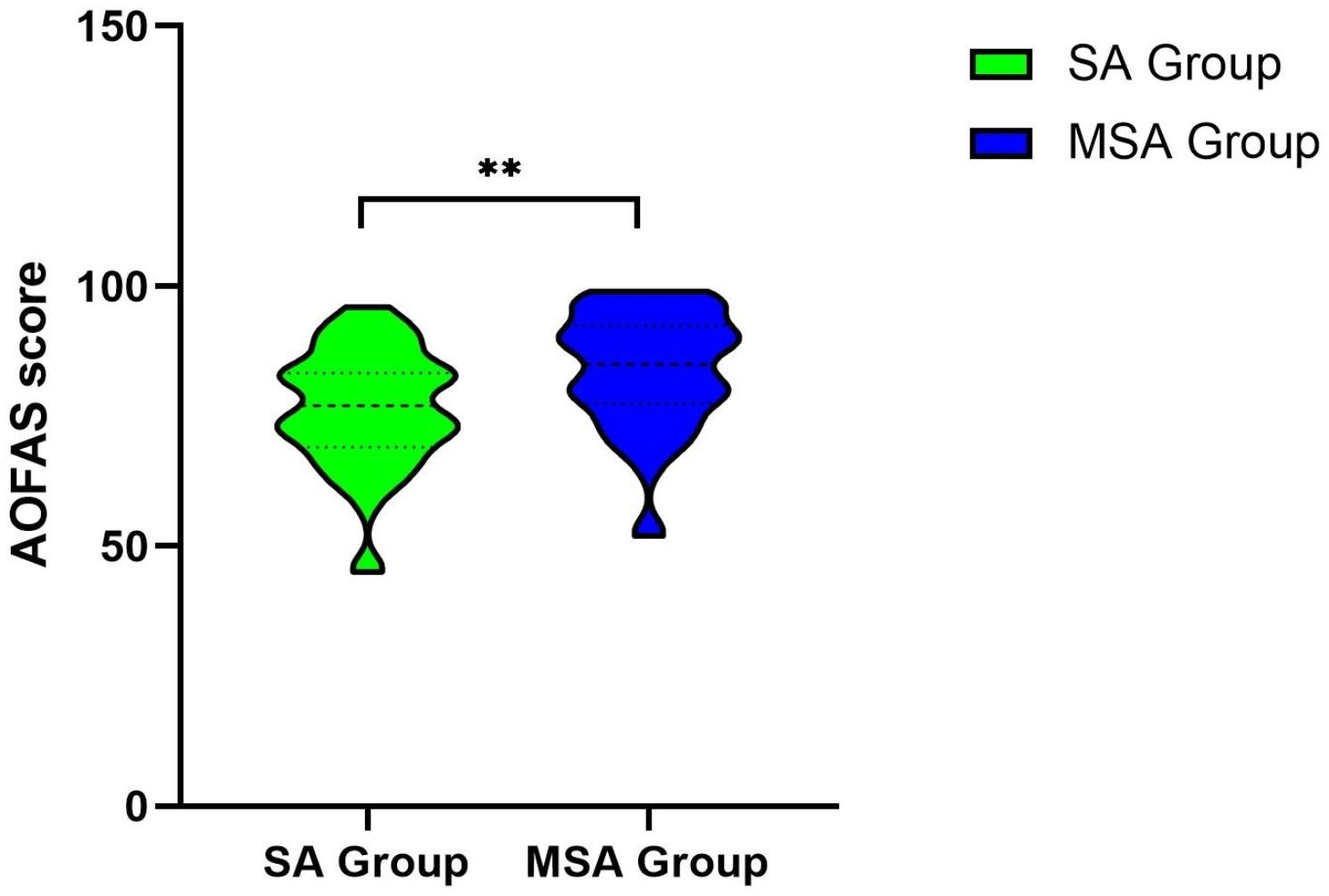
The violin figure of AOFAS scores in the two groups at final follow-up after the operation. AOFAS: The American Orthopedic Foot & Ankle Society; SA: Suture Augmentation Repair; MSA: Modified Suture Augmentation Repair. The bar indicates SD (**P < 0.01)

The mean postoperative AOFAS score of the modified suture augmentation repair group was significantly higher than that of the suture augmentation repair group (MSA group: 83.8 ± 11.3; SA group: 76.0 ± 11.3; *P* = 0.001) (Table 2; Fig. 4).

**Table 2:** Outcome characteristics

Variable	AOFAS	VAS	Anterior drawer test, %				Postoperative complications			
			Grade 0	Grade 1	Grade 2	Grade 3	Infection	Thromboses	Re-operation	Sural Nerve damage
SA group	76.3 ± 11.3	1.48 ± 0.6	0(0%)	45(90%)	5(10%)	0(0%)	3	0	0	1
MSA group	83.8 ± 11.3	1.50 ± 0.6	0(0%)	47(94%)	3(6%)	0(0%)	2	0	0	0
Differences between groups	7.5 ± 2.2	0.02 ± 0.12								
t/X2 value	3.546	0.172	0.25							
*P* value	0.001	0.863	0.617							

Data are number of patients n (%) or mean ± SD

AOFAS: The American Orthopedic Foot & Ankle Society; VAS: Visual analog scale; SA: Suture Augmentation Repair; MSA: Modified Suture Augmentation Repair; Pre-: preoperative

The mean postoperative VAS score was 1.50 ± 0.6 in the modified suture augmentation repair group and 1.48 ± 0.6 in the mean augmentation repair group. There were no statistically significant differences between the two treatment groups (*P* = 0.863) (Table 2).

There was no statistically significant difference in the postoperative anterior drawer test results between the two groups (*P* = 0.617). As shown in Table 2 and 47 (94%) patients had grade 1 laxity, and three (6%) patients had grade 2 laxity in the modified suture augmentation repair group. Simultaneously, 45 (90%) and five (10%) patients had grade 1 and grade 2 laxity, respectively.

In terms of complications, three patients with CAI had superficial wound infections and one patient had sural nerve injury in the suture augmentation repair group. Two patients in the modified suture augmentation repair group had superficial wound infections (Table 2).

## Discussion

The major contribution of this study is the proposal of a modified surgical technique for treating patients with CAI. The clinical efficacies of modified suture augmentation repair and suture augmentation repair were evaluated and compared.

Currently, a few surgical techniques for treating CAI, such as lateral ankle ligament reconstruction [19], the modified Karlsson procedure [20], modified Broström procedures [21], and arthroscopic ATFL suture augmentation repair [12], have been proposed. Although these surgical techniques have demonstrated good clinical outcomes, postoperative complications such as immunogenic reactions, infection, or recurrence are still reported to occur [19, 22]. Therefore, strategies must be developed to avoid such complications. Cordier et al. [23] demonstrated that the connectome between the lower bundle of the ATFL and CFL is sufficiently strong to transfer tension from the ATFL to the CFL. Our research team found that suturing the upper and lower tracts of the ATFL followed by suture augmentation repair not only effectively prevents the repaired ATFL from colliding with the surrounding tissues but also makes the repaired ATFL firm and stable. This surgical procedure is referred to as the modified suture augmentation repair. However, the clinical efficacy of the modified suture augmentation repair remains to be elucidated. Therefore, the clinical efficacies of suture augmentation and modified suture augmentation repairs were examined in the present study.

Similar to Tian et al. [22] and Hou et al. [11], we achieved good clinical outcomes at the final follow-up using modified suture augmentation repair in patients with CAI (AOFAS: 86.5 [Tian et al.], 85.9 [Hou et al.] vs. 83.8). Additionally, the AOFAS score increased from a baseline score of 65.9 to 83.8 in the modified suture augmentation repair group. To some extent, this finding demonstrates the feasibility and clinical efficacy of the modified suture augmentation repair technique. However, anatomical research is essential for evaluating the feasibility of the CAI surgical technique [24]. Therefore, further studies are warranted to evaluate the biomechanical and anatomical reconstruction efficacy of the modified suture augmentation repair.

In addition, we evaluated the postoperative visual analog scale (VAS) and anterior drawer test scores in the two treatment groups. Anterior drawer test scores showed no significant differences between the suture augmentation repair and modified suture augmentation repair groups. Although grade 3 laxity was not observed in all the patients after surgery, grade 2 laxity was observed in both treatment groups. This may be attributed to the patient returning to work immediately or improper recovery methods after surgery [22]. This is where we should pay attention to. No statistical difference was observed in terms of the VAS score between the two surgical techniques. In general, clinical results show that modified suture augmentation repair is feasible.

Modified suture augmentation repair is an improved technique based on suture augmentation repair. Therefore, it is not difficult for orthopedic surgeons to master this surgical technique. However, the nature of the ATFL injury in each patient is different; therefore, the selection of the surgical scheme should be based on the individual situation of the patient. Moreover, a few patients in this study had postoperative complications, such as superficial wound infections and sural nerve damage, similar to those in the study by Tian et al. [22]. It is worth considering methods to reduce the incidence of postoperative complications.

This study has some limitations. First, the follow-up time was approximately 24 months, and further follow-ups are needed as this is a newly introduced modified surgical technique. Second, this was a single-center retrospective trial with a limited number of patients. Therefore, additional multicenter-controlled trials are warranted. Nevertheless, we report promising clinical outcomes for this modified CAI surgical technique. Based on our study results, this technique may be applicable to patients with CAI for whom other treatments are not feasible. Further studies are warranted to validate this surgical technique.

## Conclusion

Both modified suture augmentation and suture augmentation repairs are good treatment options for patients with CAI. Our preliminary data indicate that superior AOFAS scores were obtained with the use of modified suture augmentation repair as compared with suture augmentation repair. This newly introduced modified surgical technique is a feasible and practical treatment option for patients with CAI.

### Electronic supplementary material

Below is the link to the electronic supplementary material.

**Supplementary Material 1:** The condition of the included patients

## Data Availability

The datasets generated and/or analysed during the current study are not publicly available due the requirements of patient privacy but are available from the corresponding author on reasonable request (Supplementary materials for the patient’s condition).

## Abbreviations

CAI: Chronic Ankle Instability
AOFAS: American Orthopedic Foot & Ankle Society
VAS: Visual Analog Scale
ATFL: anterior talofibular ligament
MSA: Modified Suture Augmentation
SA: Suture Augmentation
PSM: Propensity Score Matching
CFL: Calcaneofibular Ligament
